# Gene knockdown in HaCaT cells by small interfering RNAs entrapped in grapefruit-derived extracellular vesicles using a microfluidic device

**DOI:** 10.1038/s41598-023-30180-3

**Published:** 2023-02-22

**Authors:** Shoko Itakura, Ayaka Shohji, Sayaka Amagai, Masashi Kitamura, Kozo Takayama, Kenji Sugibayashi, Hiroaki Todo

**Affiliations:** 1 Faculty of Pharmacy and Pharmaceutical Sciences, Josai University, 1-1 Keyakidai, Sakado, Saitama 350-0295 Japan; 2 Faculty of Pharmaceutical Sciences, Josai International University, 1 Gumyo, Togane, Chiba-Ken 283-8555 Japan

**Keywords:** Drug delivery, Nanofluidics, Food nanotechnology

## Abstract

Small interfering RNAs (siRNAs) knockdown the expression of target genes by causing mRNA degradation and are a promising therapeutic modality. In clinical practice, lipid nanoparticles (LNPs) are used to deliver RNAs, such as siRNA and mRNA, into cells. However, these artificial nanoparticles are toxic and immunogenic. Thus, we focused on extracellular vesicles (EVs), natural drug delivery systems, for the delivery of nucleic acids. EVs deliver RNAs and proteins to specific tissues to regulate various physiological phenomena in vivo*.* Here, we propose a novel method for the preparation siRNAs encapsulated in EVs using a microfluidic device (MD). MDs can be used to generate nanoparticles, such as LNPs, by controlling flow rate to the device, but the loading of siRNAs into EVs using MDs has not been reported previously. In this study, we demonstrated a method for loading siRNAs into grapefruit-derived EVs (GEVs), which have gained attention in recent years for being plant-derived EVs developed using an MD. GEVs were collected from grapefruit juice using the one-step sucrose cushion method, and then GEVs-siRNA-GEVs were prepared using an MD device. The morphology of GEVs and siRNA-GEVs was observed using a cryogenic transmission electron microscope. Cellular uptake and intracellular trafficking of GEVs or siRNA-GEVs to human keratinocytes were evaluated by microscopy using HaCaT cells. The prepared siRNA-GEVs encapsulated 11% of siRNAs. Moreover, intracellular delivery of siRNA and gene suppression effects in HaCaT cells were achieved using these siRNA-GEVs. Our findings suggested that MDs can be used to prepare siRNA-EV formulations.

## Introduction

RNAi therapeutics, such as small interfering RNAs (siRNAs), which can regulate the expression of disease-related proteins by targeting mRNA, have recently become prominent in clinical applications. RNAi therapeutics are expected to be effective against diseases that are difficult to target with low molecular weight compounds or antibodies^1^. siRNAs are short double-stranded RNAs that can bind to a specific target mRNA after dissociation to single strands; thus, theoretically, siRNAs can silence the expression of mRNAs for any genes^2–4^. However, generally, siRNAs have drawbacks, such as limited membrane permeation ability and poor biostability, because they are negatively charged hydrophilic macromolecules and are easily degraded by nucleases. siRNAs must reach the cytoplasm of target cells by avoiding degradation caused by the host immune response to exhibit therapeutic effects^5^. To overcome these drawbacks, many researchers have focused on the chemical modification of siRNAs^6–8^ and making use of drug delivery systems (DDSs) to enhance biostability and achieve effective cytoplasmic delivery. Research has been performed in recent years, especially the development of artificial lipid nanoparticles (LNPs) consisting of phospholipids^9,10^. Patisiran (Onpattro), approved by FDA in 2018, was the first LNP formulation with an siRNA that can be administrated intravenously^11,12^. These LNPs were made of phospholipids, such as 1,2-Distearoyl-*sn*-glycero-3-phosphocholine (DSPC), cholesterol, polyethylene glycol, and ionic lipids, and were used for cytoplasmic delivery of siRNAs to regulate blood circulation^13^. However, LNPs have immunogenicity and low productivity due to artificial nanoparticles.

Recently, extracellular vesicles (EVs), which are natural biological LNPs secreted by various cells, have gained attention in research on DDSs. EVs have a lipid bilayer structure like liposomes and enclose a wide variety of biofunctional RNAs or proteins, such as tetraspanin, inside them or on their membranes^14,15^. They play a role in cell–cell communication related to various processes in the body, such as immune responses, viral dissemination, and tumor formation and metastasis^16,17^. Therefore, EVs are promising RNA cargo transporters because of their characteristics of carrying a variety of RNAs into recipient cells and delivering them to selected tissues^18–20^. Besides EVs derived from mammalian cells, several reports based on plant-derived ones have been published recently. Ginger-derived EVs have been reported to be mainly taken up by intestinal epithelial cells and shown to prevent colitis-associated cancer^21^. The grape exosome-like nanoparticles can be taken up by mouse intestinal stem cells and cause the induction of Lgr5^hi^ intestinal stem cells through the Wnt/β-catenin pathway^22^. Furthermore, grapefruit-derived EVs showed an anti-inflammatory effect against colitis^23^. These EVs derived from edible plants, such as fruits and vegetables, are gaining attention for their various effects on humans and are expected to be applied for the treatment of disease or improvement of health^24^.

EVs obtained from edible plants have low toxicity compared with artificial nanoparticles and are superior in terms of cost-effectiveness because large-scale preparation of such EVs is straight-forward^25^. Orally administered ginger-derived nanoparticles containing siRNA have been reported to be targeted to colon tissues^26^. Acerola-derived nanovesicles have been shown to deliver miRNA systemically via oral administration to the small intestine and liver^27^, indicating their potential for use as an oral DDS for nucleic acids in the digestive system.

Thus, understanding plant-derived EVs and taking advantage of their characteristics may lead to the development of DDS carriers capable of overcoming the problems associated with artificial nanocarriers.

The technique for loading a drug, such as an siRNA, into EVs is an important issue for using EVs as DDS carriers. The methods previously reported are classified into two different loading approaches. One approach is the endogenous method for loading cargo molecules performed by transfection into secretion cells. The other approach is an exogenous method for loading cargo forcibly by physical processing after EV secretion^20^. For loading into plant-derived EVs, methods using physical processing are required because of impracticalities associated with the endogenous method. Loading methods, such as electroporation^28^, pH gradient^29^, and sonication^30^, have been reported previously. However, these methods show low loading efficiency and cause aggregation or nonuniformity because of excessive stimulation. Although many reports have been made regarding the loading of siRNA therapeutics into EVs via electroporation^31^, found that loading efficacy was apparently overestimated because the electroporation of siRNA resulted in the formation of siRNA aggregates with the metal of the electrode. Therefore, the loading of exogenous cargoes, such as siRNA therapeutics, into EVs with a lipid bilayer membrane is still challenging. Development of highly efficient techniques for loading siRNAs is thus required.

In this study, we focused on using microfluidic technology for the preparation of EVs containing siRNA. Advances in microfabrication technology have made it possible to produce various types of microfluidic devices (MDs). MDs are used in a wide range of applications, such as flow chemistry^32^, lab-on-chip^33,34^, analytical chemistry^35^, and medicine^36^. The devices are capable of handling small volumes of liquids and rapidly carrying out reactions. The flow into a channel is stable and laminar, and the process of mixing, reacting, and purifying can be controlled. In addition, the actualization of process automation, low fabrication costs, and precision control of molecule concentrations in space and time are advantages^37^. MDs are also widely used for the preparation of LNPs in the DDS field. Chip-typed and capillary-typed devices have been employed for LNPs production^38^. LNPs with excellent controllability of their physical properties can be prepared by mixing a drug solution with an ethanolic solution of lipids in the MDs. The flow rate of the lipid solution to the drug solution makes it possible to control the particles size^39,40^. The preparation of nanoparticles containing therapeutics composed of a phospholipids^41,42^, polymers^38^, and poly-lactic-co-glycolic acid^43^ using an MD and the use of MDs for isolating EVs^44^ have been reported previously. However, the use of MDs for loading siRNAs into EVs has not been reported.

Herein, we aimed to develop a preparation technique for siRNA-containing EVs (siRNA-EVs). The potential of siRNA-EVs prepared using an MD as a siRNA carrier was evaluated. We used grapefruit-derived EVs (GEVs), which are frequently studied plant-derived EVs, and human keratinocyte cells (HaCaT cells), in which the uptake of GEVs was shown in our preliminary studies. HaCaT cells are used in this study to model cells that take up GEVs, and this technology can be applied to other cells targeted by EVs. The mixing conditions of the MD were optimized for the preparation of siRNA-GEVs by the design of experiment (DoE) and response surface method (RSM), and the physicochemical properties and knockdown effect of siRNA loaded into GEVs were evaluated.

## Results

### Collection and characterization of GEVs

**Figure 1:**
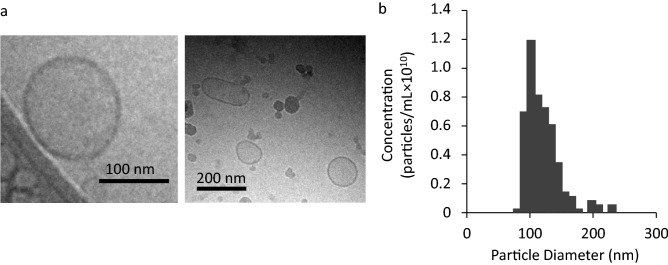
Characterization of grapefruit-derived extracellular vesicles (GEVs). (**a**) Cryogenic transmission electron microscopy (cryo-TEM) images of GEVs. (**b**) Size distribution of GEVs using a tunable resistive pulse-sensing (TRPS) technique.

GEVs were isolated and purified from juice using a standard method reported previously^45^. Figure 1a shows a morphological representation of purified nanoparticles obtained using cryo-TEM. The nanoparticles had a lipid bilayer and a single lamellar layer like the liposomes. They were identified as GEVs. The particle size, PdI, and zeta potential values of the GEVs are shown in Table 1. Figure 1b represents the particle size distribution evaluated using a tunable resistive pulse-sensing (TRPS) technique. The GEVs had weak negative charges (zeta potential, -5 mV), and their average size was approximately 120 nm.

**Table 1:** Physicochemical properties of GEVs.

	Size (nm)	PdI	Zeta potential (mV)
GEVs	122 ± 32.5	0.26 ± 0.01	−5.3 ± 0.55

The data are expressed as the mean ± S.D. (n = 3).

### Cellular uptake of GEVs into HaCaT cells

**Figure 2:**
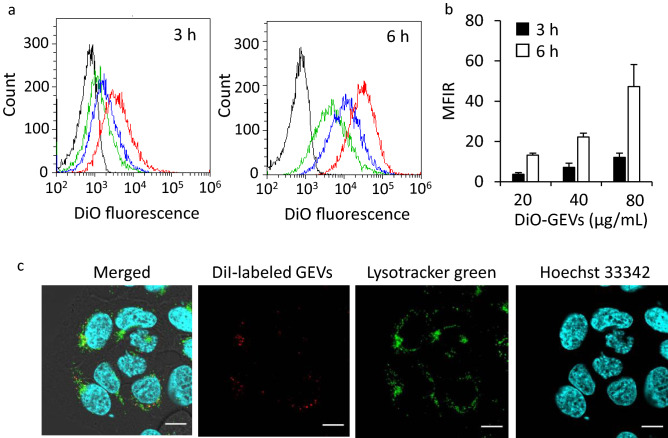
Cellular uptake and intracellular localization of GEVs. (**a**) Representative histogram showing results obtained using flow cytometry (FACS). HaCaT cells were treated with 20 (green line), 40 (blue line), and 80 µg/mL (red line) of 3,3′-dioctadecyloxacarbocyanine perchlorate (DiO)-labeled GEVs and phosphate-buffered saline (PBS; black line) for 3 and 6 h. (**b**) The mean fluorescence intensity ratios (MFIR) obtained for HaCaT cells treated with DiO-labeled GEVs. Data are presented as the mean ± S.D. (n = 3). (**c**) Representative confocal laser scanning microscopy (CLSM) images of HaCaT cells treated with 1,1′-dioctadecyl-3,3,3′,3′-tetramethylindocarbocyanine perchlorate (DiI)-labeled GEVs (red) for 6 h. Nuclei and lysosomes are stained with Hoechst 33,342 (blue) and LysoTracker DND-26 (green), respectively. Scale bars represent 10 µm.

HaCaT cells were treated with DiO-labeled GEVs for 3 and 6 h, and the uptake into HaCaT cells was determined using flow cytometry. Figure 2a,b show the histogram of DiO-positive cells and the calculated MFIR values, respectively. The cellular uptake of GEVs (20, 40, and 80 µg/mL protein concentration) increased in concentration- and time-dependent manners. In addition, the intracellular localization of GEVs was observed using CLSM. Figure 2c shows a representative CLSM image captured 6 h after the DiI-labeled GEVs were added to the HaCaT cells. Many red dots derived from DiI-labeled GEVs were colocalized with green signals, indicating endosomes/lysosomes in the cells. In addition, images of cells image treated with DiI alone as the dye control by the same method are shown in Fig. S1a. The DiIs were stained at the plasma membrane as well as the endosomal membrane and observed in many parts in the cells. Furthermore, the cellular uptake of GEVs was significantly decreased in the presence of 0.4 M sucrose, which is a clathrin-mediated endocytosis inhibitor (Fig. S1b).

### Optimization of MD conditions

**Figure 3:**
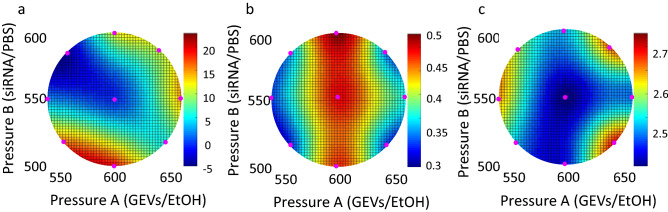
Effect of pressure on particle size, PdI, and loading efficiency of siRNA-GEVs prepared using a microfluidic method. The response surface method was applied to analyze data obtained using the design of experiment (DoE) approach. Response surface models of (**a**) mean particle size, (**b**) loading efficiency, and (**c**) PdI are shown. Pressures A and B are fluid pressures in the siRNA/PBS and GEVs/EtOH channels, respectively.

Figure 3 shows the response surface, which was drawn from the obtained data in conditions 9 and 10 to be central, for particle size (a), PdI (b), and LE% (c) as a function of pressure A and pressure B values estimated using RSM-S. Red circles in Fig. 3 show the results of the for prepared particles in the conditions shown in Table S1. Lower values were observed for the pressure around conditions 9 and 10 for particle size (a) and PdI (b), whereas a higher value was confirmed for LE%(c). The accuracy of each response surface was evaluated using leave-one-out cross-validation, which produced correlation coefficients (*r*). The *r* values of the relationships between predicted and experimental values at particle size, PdI, and LE% obtained from the cross-validation method were 0.9322, 0.4104, and 0.9752, respectively. The optimal conditions for minimum particle size and PdI, and maximum LE% were pressure A; 599 hpa and pressure B; 522 hPa by RSM-S. Moreover, the optimal solution was an estimated interval using the BS method to evaluate the accuracy of the value. When BS optimal solution deviated from the original solution and BS standard deviation was large, optimal original solution had low precision. As shown in Table 2, the optimal solution was almost the same among 1000, 2000, 3000, 4000, and 5000 times, indicating that the estimated optimal solution had high accuracy by generating the BS solution. The obtained optimal pressure condition was used to prepare siRNA-GEVs by MDs to deliver siRNAs into HaCaT cells.

**Table 2:** Optimal values of causal factors predicted using the response surface method (optimal solutions were calculated based on the bootstrap resampling with replacement at 1,000, 2,000, 3,000, 4,000, and 5,000 times).

	Pressure A Lipid/EtOH	Pressure B siRNA/PBS	Size (LOG)	PdI	Loading efficiency (%)
1000	598.7604	552.1094	2.4679	0.29464	19.3322
2000	598.8025	552.0296	2.4684	0.29537	19.2507
3000	598.7815	552.0467	2.4682	0.29539	19.3044
4000	598.7719	552.0728	2.4686	0.29538	19.2506
5000	598.7657	552.1306	2.4682	0.29525	19.2963

### Characterization of siRNA-GEVs

**Figure 4:**
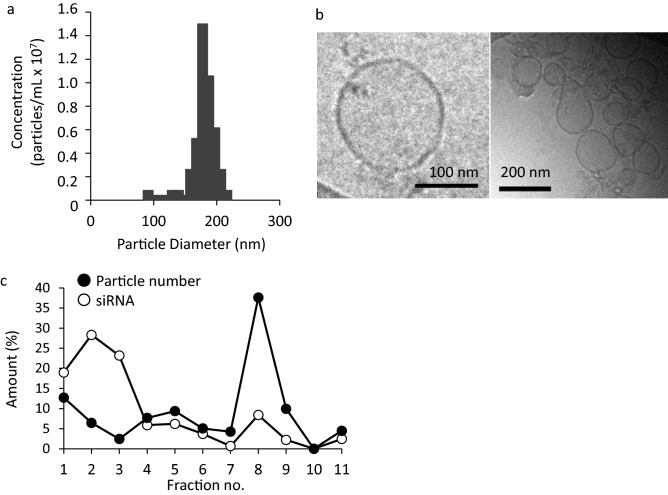
Characterization of siRNA-GEVs. (**a**) Size distribution of siRNA-GEVs using a tunable resistive pulse-sensing (TRPS) technique. (**b**) Cryogenic transmission electron microscopy (cryo-TEM) images of siRNA-GEVs. (**c**) The siRNA-GEVs prepared using a microfluidic device were fractionated by sucrose density gradient centrifugation. TAMRA-labeled siRNAs were analyzed using a fluorescence spectrophotometer, and particle numbers were calculated using qNano.

**Figure 5:**
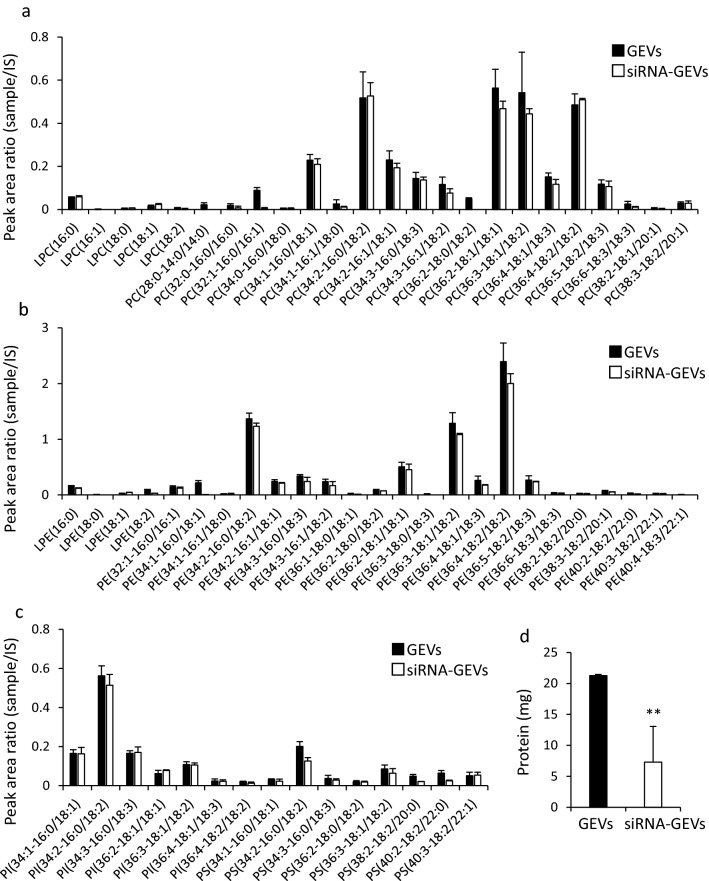
Evaluation of phospholipids and proteins in siRNA-GEVs compared with those in GEVs. (**a**) Analysis of phospholipids in GEVs and siRNA-GEVs by liquid chromatography-tandem mass spectrometry (LC–MS/MS). The data are shown as peak area ratios of (**a**) lysophosphatidylcholine and phosphatidylcholine (PC), (**b**) lysophosphatidylethanolamine and phosphatidylethanolamine (PE), (**c**) phosphatidylinositol (PI) and phosphatidylserine (PS), and (**d**) phospholipids in GEVs and siRNA-GEVs. The data are presented as the mean ± S.D. (n = 3) (**b**) The protein contents of siRNA-GEVs and GEVs were measured using BCA assay. The data are presented as the mean ± S.D., **p < 0.01 (n = 3).

Table 3 shows the particle size and zeta potential of siRNA-GEVs prepared in the optimal pressure conditions using an MD. Figure 4a shows the size distribution of siRNA-GEVs obtained using the TRPS technique. These results indicated that the particle size of siRNA-GEVs was a slightly larger (approximately 200 nm) than that of GEVs. Furthermore, siRNA-GEVs had weak negative charges similar to those of GEVs. The structure of the siRNA-GEVs observed by cryo-TEM analysis (shown in Fig. 4b) was a nanoparticle with a lipid bilayer similar to the GEV structure. Figure 4c shows the particles and siRNA distribution of siRNA-GEVs fractionated using sucrose density gradient centrifugation. Only siRNA was exhibited in fractions 1‒3, whereas siRNA coexistence with nanoparticles was observed in fractions 8 and 9, and few siRNA and particles were detected in fractions 10 and 11. Therefore, siRNA-GEVs were distributed in fractions 8 and 9. When the LE% of siRNA was measured with the samples collected from fractions 8 and 9, the LE% was 11 ± 3.1% (n = 3). Moreover, phospholipids contained in GEVs and siRNA-GEVs were determined using LC–MS/MS to investigate the effect of the preparation process of siRNA-GEVs with an MD on the change in lipid composition. This is because the loading process of siRNAs into the particles involved the reconstruction process of GEVs from their ethanol solution. Figure 5a–c show relative peak area ratios of the fatty acid types of each phospholipid to an IS. The same types of phospholipids can be compared relatively. The phospholipids identified in GEVs were lysophosphatidylcholine, PC (Fig. 5a), lysophosphatidylethanolamine, PE (Fig. 5b), PI, and PS (Fig. 5c). The same types and concentrations of phospholipids were present in siRNA-GEVs (Fig. 5a–c), although the peak area ratios of phospholipids in siRNA-GEVs appeared to be slightly decreased compared with those in GEVs. In addition, Fig. 5d shows the results of detecting the protein abundance in GEVs and siRNA-GEVs. The amount of protein in siRNA-GEVs was significantly decreased by approximately 65% compared with that in GEVs. The SDS-PAGE analysis of GEVs and siRNA-GEVs (Fig. S4) also revealed a decrease in the protein content of siRNA-GEVs.

**Table 3:** Physicochemical properties of siRNA-GEVs.

	Size (nm)	PdI	Zeta potential (mV)
siRNA-GEVs	194 ± 21.6	0.39 ± 0.04	−5.1 ± 0.53

The results were expressed as the mean ± S.D. (n = 4).

### Cellular uptake and gene knockdown effect of siRNA-GEVs in HaCaT cells

**Figure 6:**
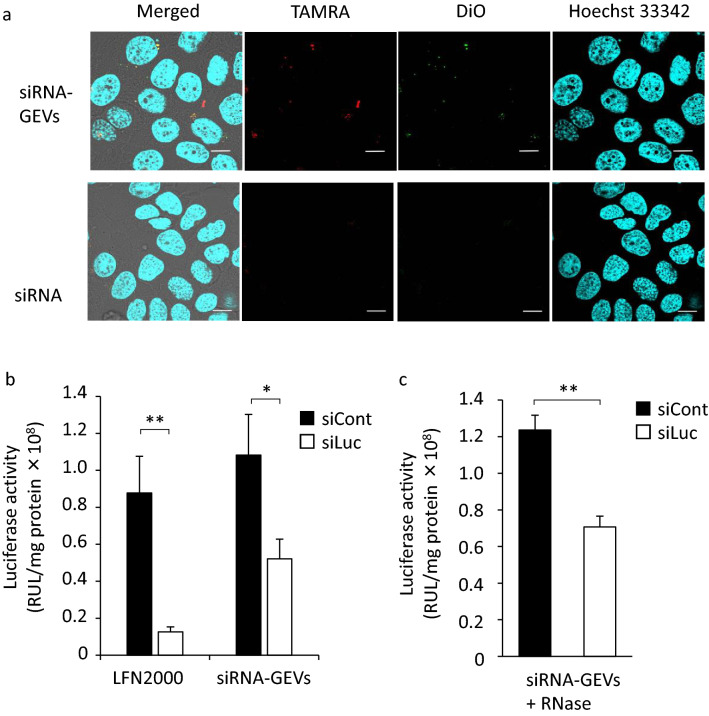
siRNA delivery and gene knockdown effect of siRNA-GEVs. (**a**) Intracellular delivery of siRNAs by siRNA-GEVs. HaCaT cells were treated with 3,3′-dioctadecyloxacarbocyanine perchlorate (DiO)-labeled GEVs (green) encapsulating TAMRA-siRNAs (red) for 6 h. Nuclei were stained with Hoechst 33,342 (blue). Scale bars represent 100 µm. (**b**) Gene knockdown effect. siRNA-GEVs were prepared using negative control siRNAs (siCont) or anti-luciferase siRNAs (siLuc). HaCaT cells were treated with 0.2 µg plasmid DNA (pDNA) encoding luciferase for 12 h, and then 10 pmol of siRNAs was added to the cells (by treating the cells with siRNA-GEVs or lipofectamine 2000). After a 24 h incubation, luciferase activity was measured. The results are presented as the mean ± S.D. *p < 0.05, **p < 0.01 (n = 3). (**c**) Gene knockdown effect of siRNA-GEVs treated with RNase. The results are presented as the mean ± S.D. **p < 0.01 (n = 3).

Figure 6a shows intracellular uptake of siRNAs from siRNA-GEVs observed using a CLSM. siRNA-GEVs were prepared using TAMRA-labeled siRNAs, and their lipid membrane was labeled with DiO. siRNAs observed as red dots were colocalized with the lipid membrane (DiO) observed in green in the cells, indicating that siRNA-GEVs were delivered into the cells. In addition, siRNAs in fractions 8 and 9 of the siRNA-GEVs were observed in HaCaT cells by sucrose density gradient centrifugation (Fig. 4a), whereas they were not observed in the other fractions (Fig. S3). Furthermore, the knockdown of luciferase activity by siRNA-GEVs was investigated to verify that the delivered siRNAs were functional. HaCaT cells, which were transfected with pcDNA encoding luciferase, were treated with siRNA-GEVs containing negative control siRNAs (siCont) or anti-luciferase siRNA (siLuc). The luciferase activity measured after incubation for 24 h is shown in Fig. 6b. Although the gene suppression ratio of siRNA-GEVs (52.7%) was inferior to that of LFN2000 (85.2%) as a commercially available reagent, siRNA-GEVs loaded with siLuc significantly suppressed luciferase expression by approximately 50% compared with siCont. In addition, the gene knockdown effect in HaCaT cells was determined by evaluating siRNA-GEVs treated with 10 µg/mL RNase A (Fig. 6c). Free siRNA was degraded by treatment with 1–20 µg/mL RNase A, as confirmed by agarose gel electrophoresis (Fig. S5). These data suggest that internalization of siRNA into GEVs exhibited gene knockdown effects.

### Effect of proteins on the uptake of GEVs in HaCaT cells

**Figure 7:**
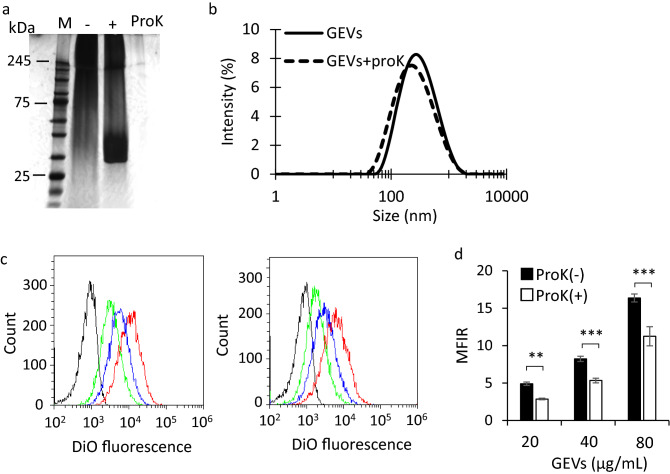
Effect of protein on cellular uptake of GEVs. (**a**) Sodium dodecyl sulfate–polyacrylamide gel electrophoresis (SDS-PAGE) of proteinase K (ProK)-treated and untreated GEV proteins. The lane of M represent molecular weight marker. Original gels are presented in Supplementary Fig. 3. (**b**) Size distribution of untreated GEVs (solid line) and ProK-treated GEVs (dotted line) by dynamic light scattering (DLS). (**c**) Representative histogram of results obtained using flow cytometry (FACS). HaCaT cells were treated with 20 (green line), 40 (blue line), or 80 µg/mL (red line) 3,3′-dioctadecyloxacarbocyanine perchlorate (DiO)-labeled GEVs (left) or PBS (black line) for 3 h. (**d**) The mean fluorescence intensity ratios (MFIR) obtained for the HaCaT cells treated with DiO-labeled GEVs. Data are presented as the mean ± S.D. **p < 0.01, ***p < 0.001 (n = 3).

As shown in Fig. 5, the phospholipid composition of siRNA-GEVs was almost the same as that of GEVs, whereas the amount of protein in siRNA-GEVs was decreased compared with that in GEVs. Because proteins on GEVs were thought to be related to cellular uptake of siRNA-GEVs, the effect of proteins on GEVs on cellular uptake was investigated. The proteins were degraded using ProK in accordance with the method reported previously^46^. SDS-PAGE analysis showed that some proteins on GEVs had disappeared after treatment with ProK (Figs. 7a, S6). As shown in Fig. 7b, minor variations were observed in the particle size distribution of GEVs and ProK-treated GEVs. Cellular uptake was evaluated using the same method as used in Fig. 2a,b after incubation of GEVs and ProK-treated GEVs in HaCaT cells for 3 h. As shown in Fig. 7c,d, cellular uptake of ProK-treated GEVs at 20, 40, and 80 µg/mL protein concentrations was significantly decreased (*p* < 0.01 for GEVs at 20 µg/mL protein concentration, *p* < 0.001 for GEVS at 40 and 80 µg/mL protein concentration) compared with that of untreated GEVs. The decrease ratio was about 30% on an average for each concentration.

## Discussion

The feasibility of loading siRNAs into plant-derived EVs, which are promising drug delivery carriers, using an MD was investigated in the present study. As reported previously by El-Andaloussi et al.^47^, approximately 25% of supplied siRNAs can be loaded into EVs by electroporation. Furthermore^29^, showed that approximately 6.5% of mRNAs were loaded by incubation. On the other hand, Kooijmans et al.^28^ described that electroporation-based loading methods are the most common approach to deliver siRNA into cells, but this method leads to aggregation of siRNAs, and the loading efficiency of siRNA was below 0.05%. We also tested whether siRNAs could be loaded into EVs via electroporation under conditions of 100–200 V, 30 ms, and 3 cycles. No siRNAs were loaded into GEVs because of their strong aggregation (data not shown). Thus, reported methods are insufficient in terms of reproducibility and operability. The siRNA loading efficiency with an MD was approximately 11% in this study, and intracellular delivery of siRNA and gene suppression were achieved in HaCaT cells (Figure 6a,b). The siRNA loading efficiency was similar to previous reports and not so high, although the MD method can produce and scale up easily compared with the electroporation method as described in the Introduction. These results suggested that loading siRNA to EVs with an MD may be a promising method in addition to conventional approaches, such as electroporation. Another advantage for particle preparation with an MD is the precision control of flow rate in microfluidic channels. Therefore, this technique can enable precise control of particle size. Hashiba et al.^39^ reported that the particle size and PdI were important parameters for the transfection activity of mRNA-loaded LNPs. Therefore, optimal preparation conditions for preparing siRNA-loaded EVs were investigated using a DoE approach.

The GEVs taken up into HaCaT cells were colocalized with endosomes/lysosomes according to the CLSM observations (Fig. 2c). In addition, sucrose, which is a clathrin-mediated endocytosis inhibitor, decreased cellular uptake of GEVs in the HaCaT cells (Fig. S1b). These results suggest that the endocytosis pathway played a role in the intracellular delivery of GEVs and the clathrin-mediated endocytosis pathway was involved in the cellular uptake. The GEVs have lipids, proteins, and other components, and their uptake may have an effect on the physiology of keratinocytes. Reports of grapefruit-derived EVs have indicated that they are efficiently uptaken by various tumor cells and immune cells^23,48^; however, this is the first report indicating that they are taken up in HaCaT cells and human keratinocytes. The physiological activity of GEVs in keratinocytes has not been investigated yet, although such work would be applicable for therapeutic nanoparticles and DDS carriers.

The major components that affected the physical properties of the EV surface were phospholipids and proteins, both of which are known to be involved in the intracellular uptake of EVs^49^. Therefore, it is important to investigate components, such as phospholipids and proteins in siRNA-GEVs, to consider the effect of siRNA-GEVs on drug delivery. siRNA-GEVs prepared using an MD had increased particle size but little change in lipid composition (Fig. 5a–c). These results suggested that phospholipids in GEVs were mostly used to construct siRNA-GEVs. However, approximately 50% of proteins in GEVs were lost during the preparation process of siRNA-GEVs (Fig. 5d). In the presence of ethanol during the siRNA-GEV preparation process, proteins that are insoluble in ethanol would not be involved in the construction of nanoparticles with an MD. This is a drawback for preparation methods using an MD; however, the modification of particles may be possible by the addition of functional materials to the ethanol solvent.

siRNAs loaded in GEVs were taken up into cells (Fig. 6a) and suppressed luciferase activity (Fig. 6b). These results suggested that phospholipids in GEVs are associated with siRNA delivery. On the other hand, it was reported that the cellular uptake of exosomes derived from SKOV3 cells was decreased by treatment with ProK, indicating that proteins of exosomes had an important role in intracellular delivery^50^. In this study, as shown in Fig. 7d, treatment with ProK significantly decreased the cellular uptake of GEVs in HaCaT cells, but 70% uptake was maintained. This indicated that phospholipids as well as proteins may be important for the cellular uptake of GEVs, considering that the cellular uptake efficiency was maintained at a high level compared with the decrease in protein content.

Phospholipid analysis revealed that PC and PE were detected a high levels in GEVs and siRNA-GEVs (Fig. 5b,c)^51^, reported that PC and PE were abundant as phospholipids in GEVs, which were also the most frequently detected lipids in this study. They described that PC is an important lipid in building the surface of EVs, and it is involved in the selective uptake of GEVs into enterobacteria. On the other hand, PE is located on the inside of the lipid bilayer in EVs and exerts a negative curvature effect against the lipid bilayer by forming an inverted hexagonal structure because of its small hydrophilic group^49,52^ reported that a correlation can be made between the shape of the lipids included in the facing monolayers of two contacting membranes and the occurrence of fusion. During a fusion process, a lipid with negative curvature favors the incorporation of lipids. This characteristic may play an important role in promoting the endosomal escape and intracellular delivery of siRNAs^52^. Therefore, siRNA-GEVs exhibited a gene knockdown effect, but the effect was lower than with LFN2000 (Fig. 6b). Improvement in the restoration of proteins is a future issue, making it possible to use EVs as a DDS for siRNAs with greater efficiency. Herein, the change in phospholipids and protein content of siRNA-GEVs prepared by the MD method were investigated. This will provide significant insights for the construction of siRNA-loaded EVs.

## Conclusion

The technique for loading siRNAs into EVs is still challenging. In this study, siRNAs were loaded into GEVs with 11% efficiency. siRNA-GEVs were fabricated by optimizing pressure conditions in the MD. Moreover, the gene suppression effect of siRNA-GEVs was demonstrated using HaCaT cells. It is known that proteins and phospholipids in GEVs affect the cellular uptake of GEVs. In the present study, proteins were removed when siRNA-GEVs were prepared using an MD, which might have affected the cellular uptake of siRNA-GEVs. These results may provide a new method and information for loading siRNAs into EVs, which are promising natural DDSs, by using an MD.

## Materials and methods

### Materials

HaCaT cells were purchased from Cell Line Service GmbH (Eppelheim, Germany). Dulbecco’s modified Eagle’s medium, trypsin–EDTA solution, and RIPA buffer were purchased from FUJIFILM Wako Pure Chemical Corporation (Osaka, Japan). Fetal bovine serum (FBS) was obtained from Sigma Aldrich (MO, USA). Negative control siRNAs (21-mer; 5′-CUUACGCUGUCAUGAUCGAtt-3′; 5′-UCGAUCAUGACAGCGUAAGtt-3′), anti-luciferase siRNAs (21-mer; 5′-CUUACGCUGAGUACUUCGAtt-3′; 5′-UCGAAGUACUCAGCGUAAGtt-3′), and TAMRA-labeled siRNAs (21-mer; 5′-TAMRA-UAUUGCGUCUGUACACUCAtt-3′; 5′-TAMRA-UGAGUGUACAGACGCAAUAtt-3′) were synthesized by Fasmac Co., Ltd. (Kanagawa, Japan). Luciferase-pcDNA3 was obtained from the William Kaelin Lab (Addgene; plasmid #18,964). Furthermore, 3,3′-dioctadecyloxacarbocyanine perchlorate (DiO) and 1,1′-dioctadecyl-3,3,3′,3′-tetramethylindocarbocyanine perchlorate (DiI) were obtained from Promo Cell GmbH (Heidelberg, Germany). SYBR GOLD, LysoTracker™ Green DND-26, Hoechst 33342, and Pierce™ Silver Stain Kit were purchased from Thermo Fisher Scientific (MA, USA). Proteinase K (ProK) was obtained from Kanto Chemical Co., Inc. (Tokyo, Japan).

### Isolation and characterization of GEVs

Commercially available grapefruits (grown in South Africa) were used in this study. The grapefruits were assigned a voucher no. Experimental of plants, including the collection of plant material, comply with relevant institutional, national, and international guidelines and laws. The grapefruit juice used for GEVs collection was prepared using the one-step sucrose cushion ultracentrifugation method^45^. Specifically, grapefruit juice was centrifuged at 3,000 × *g* for 10 min, followed by centrifugation at 10,000 × *g* for 30 min. The supernatant was filtered through a 0.22-µm filter. For sucrose cushion-based isolation, 40 mL of the filtrate was loaded over 4 mL of 30% sucrose in phosphate-buffered saline (PBS) and centrifuged at 141,000 × *g* for 90 min at 4 °C using a Himac CP80WX machine with swing rotor 28S (Eppendorf Himac Technologies Co., Ltd, Ibaraki, Japan). The supernatant was discarded, and the sucrose layer was resuspended in PBS, followed by ultracentrifugation at 141,000 × *g* for 90 min at 4 °C to wash and pellet the GEVs. The pellet was resuspended in PBS and ultracentrifuged at 16,500 × *g* for 10 min at 4 °C. The size of GEVs was measured using qNano, which is a TRPS technique (Meiwaforsis, Tokyo, Japan). The PdI and zeta potentials of the GEVs were measured using a Zetasizer Nano ZS (Malvern Panalytical Ltd, Worcestershire, UK). The protein concentration of the GEV solution was determined using a BCA protein assay kit (FUJIFILM Wako Pure Chemical Corporation).

### Preparation of siRNA-GEVs using an MD

A 5 Input 3D Chip (150 nm) and a P-pump (Dolomite, Blacktrace Holdings Ltd., Royston, UK) were used as the MD (Fig. S2). Because the pump used in this study was of the non-pulsating type, stable liquid feeding can be maintained. Approximately 20 µg/mL (protein concentration) GEVs in ethanol and 400 nM siRNAs in PBS were injected into channels A and B, respectively, as shown in Fig. S2. The pressure conditions of pump A and pump B were selected on the basis of a DoE built using a central composite design, as shown in Table S1. GEVs and siRNAs were injected into the microchannels using 10 conditions. The particle size and PdI were evaluated using a Zetasizer Nano, dynamic light scattering (DLS) technique. To evaluate siRNA loading efficiency (LE%), the quantity of siRNAs was calculated by analysis of fluorescence intensity. The siRNAs were stained using SYBR Gold after ultrafiltration using a Nanosep device (10 kDa MWCO; Pall Corporation, NY, USA).

### Optimization of microfluidics conditions

The conditions of sending pressure of pump A and pump B were optimized using the DoE and RSM^53,54^. The optimal preparation conditions and their credible ranges were estimated using the RSM incorporating thin-plate spline interpolation (RSM-S) and bootstrap (BS) resampling^53,54^, which was performed using dataNESIA (version 3.0, Azbil Corp., Tokyo, Japan).

The optimal solution was to obtain preparation conditions that minimized particle size and PdI and maximized LE%. The accuracy of the optimal solution was confirmed by valuation of the BS optimal solution and standard division. The frequency numbers of resampling with replacements were set at 1000, 2000, 3000, and 4000.

### Cryo-transmission electron microscopy

The morphology of GEVs and siRNA-GEVs was observed using a cryogenic transmission electron microscope (cryo-TEM). Nanoparticles (1 μL of 5 mM lipid concentration) were applied to hydrophilized copper grids (200 mesh; JEOL Ltd., Akishima, Tokyo, Japan) and blotted. The samples were rapidly frozen using a rapid freezing system (EM-CPC, Leica Microsystems, Tokyo, Japan) and then observed using an EM-3100FEF cryo-TEM (JEOL Ltd.) at an accelerating voltage of 300 kV. Observations were performed using a cooling holder (model 626; Gatan, CA, USA).

### Evaluation of cellular uptake of nanoparticles

The GEVs were labeled with DiO (Ex: 484 nm, Em: 501 nm). In brief, 2 µL of the 500 µM DiO-ethanol solution was added to the GEV suspension containing 50 µg of protein. The suspension was incubated for 10 min at room temperature, followed by washing by ultracentrifugation to remove unbound dye. Approximately 4 × 10^4^ HaCaT cells/well were seeded in 24-well plates. After overnight incubation at 37 °C, the cells were treated with 20, 40, or 80 µg/mL of DiO-labeled GEVs for 3 or 6 h at 37 °C. To investigate the effect of endocytosis on cellular uptake, cells were treated with FITC-labeled transferrin (20 µg/mL) and DiO-labeled GEVs (40 µg/mL) in the presence of 0.4 M sucrose, which is a clathrin-mediated endocytosis inhibitor. Transferrin is a protein taken up by clathrin-mediated endocytosis. The cellular uptake of GEVs was evaluated using a flow cytometer (CytoFLEX, Beckman Coulter, Inc., CA, USA). The mean fluorescence intensity ratio (MFIR) was calculated by dividing the fluorescence intensity obtained from the sample by that obtained from the untreated cells.

### Evaluation of intracellular trafficking of GEVs or siRNA-GEVs

The GEVs were labeled with DiI (Ex: 550 nm, Em: 565 nm), as described above. Approximately 2.0 × 10^5^ HaCaT cells were seeded in a Poly-L-Lysine-coated glass-bottomed dish (Matsunami, Osaka, Japan). After overnight incubation at 37 °C, the cells were treated with 40 µg/mL of DiI-labeled GEVs for 6 h at 37 °C. Endosomes/lysosomes and nuclei were stained using LysoTracker™ Green DND-26 (Ex: 504 nm, Em: 511 nm) and Hoechst 33,342 (Ex: 352 nm, Em: 461 nm), respectively. Cellular images were observed using a confocal laser scanning microscope (CLSM) FV-3000 (Olympus, Tokyo, Japan) equipped with an oil-immersion objective lens (Plan-Apochromat 63x/NA 1.4). Moreover, to investigate the intracellular localization of the siRNA-GEVs, TAMRA-labeled siRNAs and lipids were labeled with DiO, as described above. Hoechst 33,342, LysoTracker™ Green DND-26, and DiO-TAMRA were excited by 405, 488, and 561 nm laser wavelengths, respectively.

### Sucrose density gradient fractionation

siRNA-GEVs were prepared using TAMRA-labeled siRNAs, as described above. The labeled siRNA-GEVs were applied to a discontinuous sucrose density gradient (5–60%) and then ultracentrifuged at 220,000 × *g* for 2 h at 4 °C using a Himac CS100GXL machine with a swing rotor S55S (Eppendorf Himac Technologies Co., Ltd, Ibaraki, Japan). As a result, 11 fractions were obtained. Sucrose was removed by dialysis using 10,000 MWCO Slide-A-Lyzer dialysis cassettes (Thermo Fisher Scientific) at 4 °C overnight. The fluorescence emitted by TAMRA-siRNAs was measured using a fluorescence spectrophotometer (FP8300, JASCO Corporation, Tokyo, Japan) set at an excitation wavelength of 565 nm and fluorescence wavelength of 580 nm. The particle numbers for all the fractions were measured using qNano (Meiwaforsis, Tokyo, Japan).

### Phospholipid analysis by liquid chromatography-tandem mass spectrometry (LC–MS/MS)

To analyze the lipid components in GEVs and siRNA-GEVs, the LC–MS/MS Method Package for Phospholipid Profiling (Shimadzu Corporation, Kyoto, Japan) was used, according to the manufacturer’s instructions. The library of phospholipid targets in the method package includes phosphatidylcholine (PC), phosphatidylethanolamine (PE), phosphatidylglycerol (PG), phosphatidylinositol (PI), phosphatidylserine (PS), and sphingomyelin (SM). Briefly, 1.0 × 10^11^ particles of GEVs and siRNA-GEVs (prepared using GEV fractions with the same particle number) were added to methanol containing 0.1% formic acid for mass spectral analysis. Next, 5 µL of the sample solution was injected into a Kinetex C8 column (internal diameter 2.1 mm, length 150 mm, and size 2.6 µm; Phenomenex) at a flow rate of 0.3 mL/min. The samples were eluted using a gradient of mobile phases A (20 mM ammonium formate in water) and B (isopropanol: acetonitrile = 1:1 v/v). The concentration of mobile phase B was programmed at 20% (0 min)–20% (1 min)–40% (2 min)–92.5% (25 min)–92.5% (26 min)–100% (35 min)–20% (38 min). The oven temperature was 45 °C. Data processing and lipid identification/quantification were performed using LabSolutions software (version 5.99 SP2; Shimadzu Corporation, Kyoto, Japan). The analytical results were obtained by multiple reaction monitoring of transitions. The peak area ratio was calculated by dividing the area of the peak obtained for the sample by that obtained for the internal standard (IS); 17:0–20:4 PI (Avanti Polar Lipids) was added as an IS to each sample to obtain a final concentration of 0.38 µmol/L.

### ProK treatment

ProK treatment was carried out according to the previously reported method ^46^. GEVs (250 µg/mL) were treated with ProK (50 µg/mL) for 30 min at 37 °C, and then the GEVs were incubated with 5 mM phenylmethylsulfonyl fluoride (PMSF) for 10 min at 37 °C.

### Evaluation of protein by SDS-PAGE

The protein contents of GEVs and siRNA-GEVs were evaluated by sodium dodecyl sulfate–polyacrylamide gel electrophoresis (SDS-PAGE). First, 100 µg of GEVs and siRNA-GEVs was condensed by ultracentrifugation. The pellets were dissolved in RIPA buffer and the solutions were centrifuged at 21,500 × *g* for 10 min at 4 °C. The supernatants were mixed with 6 × sample buffer containing 2-mercaptoethanol and incubated at 95 °C for 4 min. Next, the samples were loaded on 12% polyacrylamide gels (TGX™ FastCast™ Acrylamide kit, Bio-Rad Laboratories, CA, USA). Electrophoresis was performed at 150 V, and then the gels were silver-stained (Pierce™ Silver Stain Kit). Protein Ladders covering 10–180 kDa ranges (NIPPON Genetics Co., Ltd.) were loaded.

### Gene knockdown assay

HaCaT cells were seeded at 2.0 × 10^4^ cells/well in 24-well plate and incubated at 37 °C overnight. The cells were transfected with luciferase-pcDNA3 (0.2 µg) in the presence of lipofectamine 2000 (LFN2000) and incubated at 37 °C for 12 h. Next, the cells were washed with PBS. siRNA-GEVs (containing 10 pmol siRNAs) were added and then the cells were incubated for 24 h at 37 °C. After incubation, luciferase activity was evaluated using the luciferase assay system (Promega, WI, USA) in accordance with the manufacturer’s protocol. The luminescence intensity was detected (by compensating for the protein concentration) using a microplate reader (Synergy H1, Agilent Technologies, CA, USA). The gene knockdown effect was evaluated after treatment with 10 µg/mL RNase A (NIPPON GENE CO., LTD. Tokyo, Japan) at 37 °C for 30 min using the same methods as described above.

### Statistical analysis

Statical analysis was performed using ANOVA and Dunnett test with JMP Pro version 16.0.0 (SAS Institute, Cary, NC, USA). The reported *p*-values were considered statistically significant at *p* < 0.05.

## Supplementary Information

Supplementary Information.

## Data Availability

The datasets used in the current study available from the corresponding author on reasonable request.
