# Supplementary material for: Gene knockdown in HaCaT cells by small interfering RNAs entrapped in grapefruit-derived extracellular vesicles using a microfluidic device
Source: Sci Rep. 2023 Feb 22;13:3102. doi: 10.1038/s41598-023-30180-3 (PMC9947018; doi:10.1038/s41598-023-30180-3)
Supplement: Supplementary file 1 — Supplementary Information. [file 41598_2023_30180_MOESM1_ESM.pdf]

Supplementary Information

Gene knockdown in HaCaT cells by small interfering RNAs entrapped in grapefruit-derived extracellular vesicles using a microfluidic device

Shoko Itakura[1], Ayaka Shohji[1], Sayaka Amagai[1], Masashi Kitamura[1], Kozo Takayama[1], Kenji Sugibayashi[1,2], Hiroaki Todo[1,*].

[1]Faculty of Pharmacy and Pharmaceutical Sciences, Josai University, 1-1 Keyakidai, Sakado, Saitama 350-0295, Japan.

[2]Faculty of Pharmaceutical Sciences, Josai International University, 1 Gumyo, Togane, Chiba-ken 283-8555, Japan.

[*]Corresponding author: Hiroaki Todo

E-mail: ht-todo@josai.ac.jp

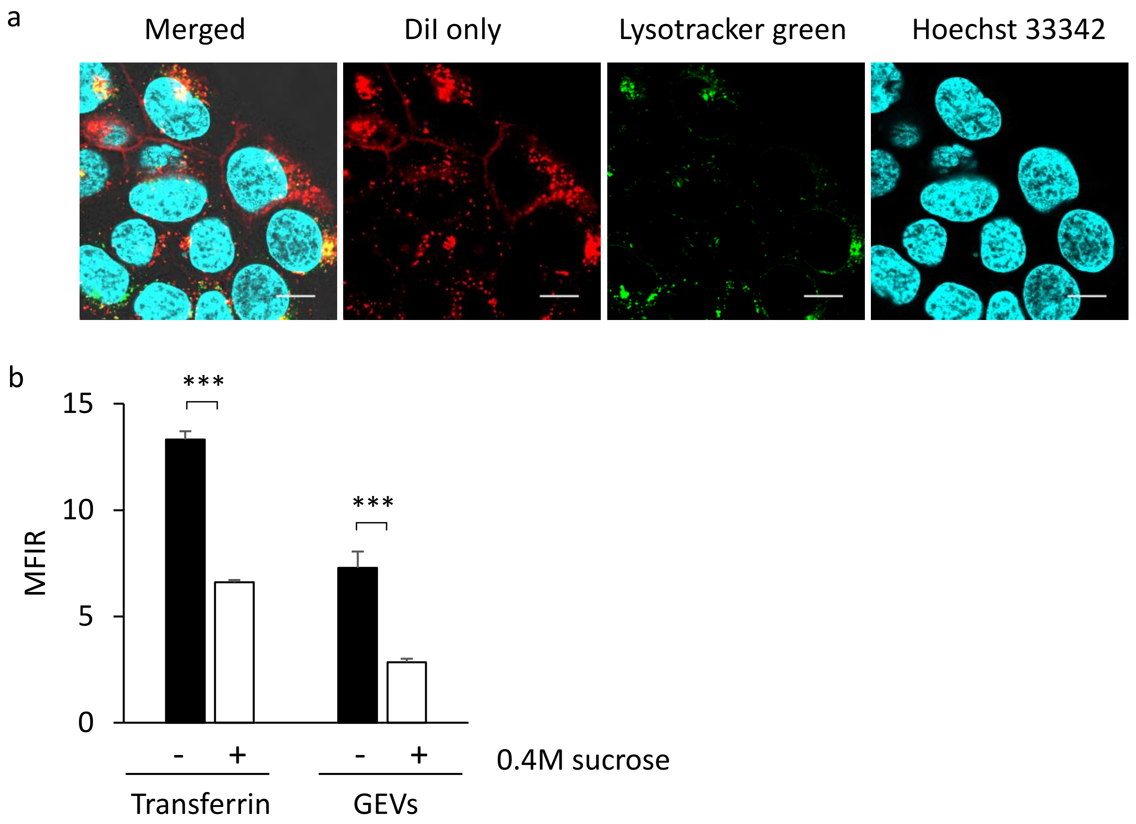

Figure S1. Cellular uptake of GEVs.
(a) Representative confocal CLSM images of HaCaT cells treated with DiI solution (red) for 6 h. Nuclei and lysosomes are stained with Hoechst 33342 (blue) and LysoTracker DND-26 (green), respectively. Scale bars represent 10 µm. (b) Effect of endocytosis inhibitor on cellular uptake of GEVs. HaCaT cells were treated with FITC-labeled transferrin and DiO-labeled GEVs in the presence or absence of 0.4 M sucrose, which is a clathrin-mediated endocytosis inhibitor.

Table S1. Pressure of A and B conditions determined using the central composite design.

Conditions	Pressure A (hPa)	Pressure B (hPa)
1	600	600
2	585	635
3	550	650
4	515	635
5	500	600
6	515	565
7	550	550
8	585	565
9, 10	550	600

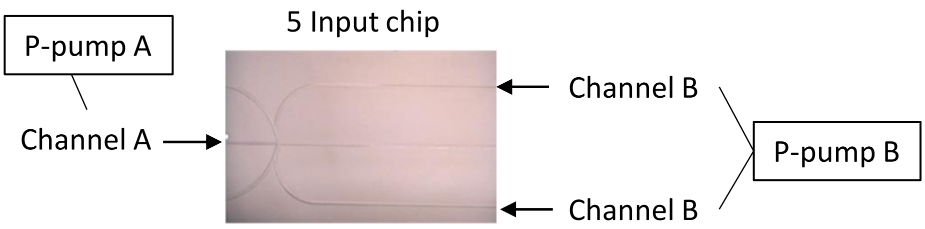

Figure S2. Microfluidics system used in this study.

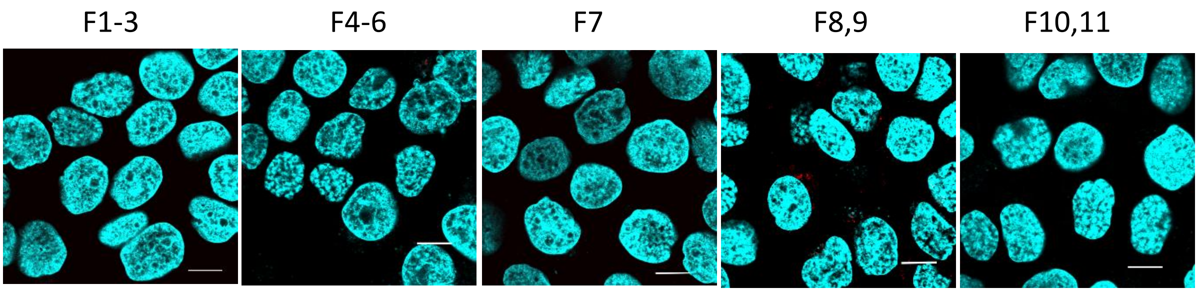

Figure S3. CLSM images of HaCaT cells treated with the fraction in Figure 4a.
HaCaT cells were treated with the fraction of siRNA-GEVs by sucrose density gradient centrifugation in Figure 4a for 6 h. Red and blue indicate TAMRA-siRNAs and nuclei stained with Hoechst 33342, respectively. Scale bars represent 100 µm.

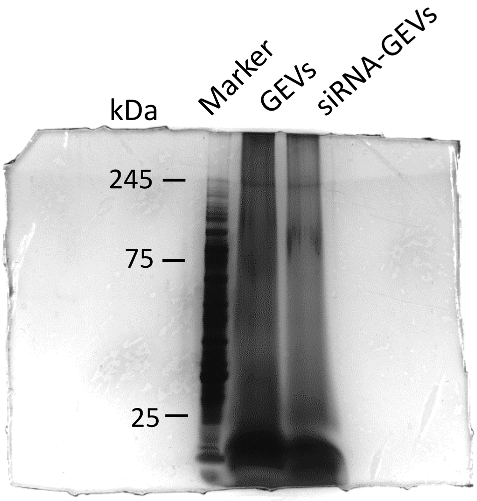

Figure S4. SDS-PAGE image of proteins from GEVs and siRNA-GEVs.

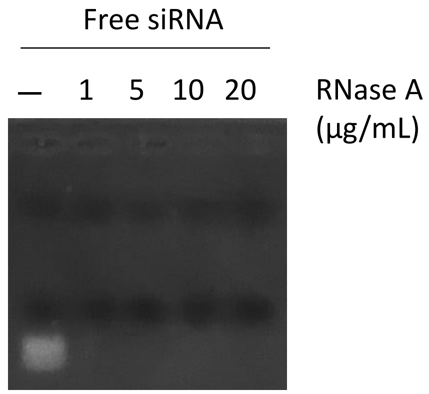

Figure S5. Image of 2% agarose gel electrophoresis of siRNA treated with RNase A. siRNAs were incubated with RNase A at the indicated concentration at 37 °C for 30 min.

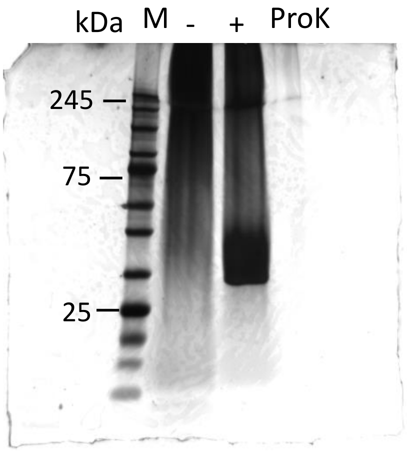

Figure S6. Original SDS-PAGE image of Figure 7a.
